# Recommendations for Health Equity and Virtual Care Arising From the COVID-19 Pandemic: Narrative Review

**DOI:** 10.2196/23233

**Published:** 2021-04-05

**Authors:** James Shaw, LaPrincess C Brewer, Tiffany Veinot

**Affiliations:** 1 Institute for Health System Solutions and Virtual Care Women's College Hospital Toronto, ON Canada; 2 Joint Centre for Bioethics University of Toronto Toronto, ON Canada; 3 Mayo Clinic Rochester, MN United States; 4 School of Information School of Public Health University of Michigan Ann Arbor, MI United States

**Keywords:** virtual care, health equity, health disparities, health informatics, COVID-19, telemedicine, telehealth, digital health

## Abstract

**Background:**

The COVID-19 health crisis has disproportionately impacted populations who have been historically marginalized in health care and public health, including low-income and racial and ethnic minority groups. Members of marginalized communities experience undue barriers to accessing health care through virtual care technologies, which have become the primary mode of ambulatory health care delivery during the COVID-19 pandemic. Insights generated during the COVID-19 pandemic can inform strategies to promote health equity in virtual care now and in the future.

**Objective:**

The aim of this study is to generate insights arising from literature that was published in direct response to the widespread use of virtual care during the COVID-19 pandemic, and had a primary focus on providing recommendations for promoting health equity in the delivery of virtual care.

**Methods:**

We conducted a narrative review of literature on health equity and virtual care during the COVID-19 pandemic published in 2020, describing strategies that have been proposed in the literature at three levels: (1) policy and government, (2) organizations and health systems, and (3) communities and patients.

**Results:**

We highlight three strategies for promoting health equity through virtual care that have been underaddressed in this literature: (1) simplifying complex interfaces and workflows, (2) using supportive intermediaries, and (3) creating mechanisms through which marginalized community members can provide immediate input into the planning and delivery of virtual care.

**Conclusions:**

We conclude by outlining three areas of work that are required to ensure that virtual care is employed in ways that are equity enhancing in a post–COVID-19 reality.

## Introduction

### Background

The COVID-19 health crisis has disproportionately impacted populations who have been historically marginalized in health care and public health, including low-income and racial and ethnic minority groups [1]. In the state of Louisiana in the United States, Black patients made up 59% of deaths related to COVID-19 in the early months of the pandemic despite representing only 33% of the state’s population [1]. These disparities in COVID-19 outcomes between Black Americans and White Americans can be observed at a population-wide level [2], and Hispanic and Indigenous American communities have also been disproportionately affected by COVID-19 [3,4]. In the United Kingdom, communities with higher concentrations of racial and ethnic minority groups and lower average income have been more likely to have a higher concentration of COVID-19 cases [5]. Outbreaks of COVID-19 have also been documented in homeless shelters in the United States and Canada [6-8], illustrating the challenges in combating the spread of the disease in congregate living settings and especially among under-resourced communities. In addition to the well-documented impact of COVID-19 on vulnerable older adults living in long-term care settings [9], these data demonstrate that COVID-19 has disproportionately affected groups who face systematic barriers to care.

The primary strategy for maintaining access to ambulatory and outpatient health services during the pandemic has been to rapidly virtualize, creating systems of health care that rely on telephone visits, video visits, and methods of asynchronous communication such as email, SMS text message, and patient portal messages [10]. Although there are multiple terms referring to the use of information and communication technologies to deliver health services [11], in this paper we refer to applications of this collection of technologies in health care as “virtual care.” A number of contributions have already been published since the onset of the pandemic proposing strategies to ensure that virtual care technologies do not exacerbate disparities in access to health care and health outcomes [12-15]. A large body of literature illustrates how relying on health-related digital technologies can enhance existing inequities—for example, where people from low-income communities are unable to access needed primary care appointments [16].

We make two primary contributions in this paper. Our first contribution is to summarize literature published in 2020 that is explicitly focused on recommending strategies to promote health equity in the delivery of virtual care in the context of the COVID-19 pandemic. We produce a synthesis of these recommendations and organize them according to the three levels at which they are most relevant: (1) policy and government, (2) organization and health system, and (3) community and patient. Our second contribution is to specifically highlight three strategies arising from this literature that are immediately practical and often neglected in the implementation of virtual care initiatives. We conclude by outlining what we view as the central considerations on which governments and health system leaders will need to focus to ensure virtual care is equitable in a sustainable way after COVID-19. We start by reviewing the concept of the digital divide and its links with virtual care in the context of COVID-19.

### The Digital Divide

The concept of the digital divide has been widely discussed in the social science literature; this work outlines three “levels” of the divide that are central to understanding the relationships between social inequalities and information technologies [17]. Although originally used narrowly to refer to the gap between those who had access to technologies and those who did not (the “first-level divide”), the concept of the digital divide has evolved to include disparities in technology literacy (the “second-level divide”) and disparities in outcomes (the “third-level divide”) related to technology use [18]. Specifically, the second-level divide refers to the fact that although some individuals might have access to the internet and digital devices, they might not have the skills and knowledge necessary to use these technologies effectively. The third-level divide indicates that even where some individuals might have sufficient knowledge of how to use the technologies, they might not be able to convert their use of technologies into outcomes that improve their lives. The growing presence of information and communication technologies in health care over the past several years has led to growing attention to the implications of the digital divide for access to, and outcomes of, health care services involving such technologies [19,20].

Reviews of the digital divide in health care have illustrated some important considerations about the role of technologies in either increasing or decreasing disparities in health and health care. Weiss et al (2019) [16] explained that understanding the impact of a health-related technology on health disparities is context-dependent, and requires close attention to particular groups of users and their pathways of access and use. In addition, insights are accumulating regarding strategies to enhance access to, and use of, technologies for health care. A 2017 review of the literature outlined strategies related to increasing access, universal technology design, cultural sensitivity, and efforts to promote participation among underserved groups, all of which are promoted as strategies to reduce technology-related health disparities [19]. We now turn to reviewing applications of this body of knowledge to the COVID-19 pandemic that have been published since the onset of the pandemic in early 2020.

## Methods

We completed a narrative review of literature published in 2020 focused on recommendations for the promotion of health equity in the delivery of virtual care as a result of the COVID-19 pandemic. Drawing on methodological guidance for narrative literature reviews related to clarity of focus and transparency of search strategy [21,22], our review focused explicitly on literature that was published in direct response to the widespread use of virtual care during the COVID-19 pandemic, and had a primary focus on providing recommendations for promoting health equity in the delivery of virtual care. Fit with these two criteria formed the inclusion criteria. We did not assess articles for quality, and included all those articles that met the two inclusion criteria. Informed by recent discussions of quality in narrative reviews [21], we provide a detailed representation of our search strategy in Table 1.

**Table 1:** Literature search strategy.

Search method	Search details	Number of included papers
MEDLINE	Search in title, abstract, keywords, and subject headings Search limited to year 2020 Search string: [(healthcare ADJ dispar*) OR (“health care” ADJ dispar*) OR (health ADJ dispar*) OR (health ADJ equit*)] AND [“virtual care” OR “digital health” OR “telemedicine” OR “telehealth”]	Total number of results: 43 Number excluded on screening (not relevant based on fit with inclusion criteria): 35 Total included papers: 8
Google Scholar	General search in Google Scholar Search limited to year 2020 Search string: (“healthcare disparities” OR “health disparities” OR “health equity“) AND (“virtual care” OR “digital health” OR “telemedicine” OR “telehealth”)	Total number of results (first 5 pages): 50 Number excluded on screening (not relevant based on fit with inclusion criteria): 45 Total included papers: 5
Forward and backward reference searching	Identify highly relevant papers cited by included papers Identify highly relevant papers that cite included papers	Total included papers: 2

We selected for inclusion only those publications that met the above two criteria, and extracted information on the following: (1) the framing of the paper, (2) the challenges each paper identified in relation to health equity and virtual care, and (3) the recommendations provided to promote health equity through virtual care. We then classified recommendations according to three levels of intervention: (1) policy and government, (2) organization and health system, and (3) community and patient. We use the findings from this literature search to highlight three important practical strategies that require attention and are at high risk of being overlooked, and then comment on strategies to make equitable virtual care sustainable following the COVID-19 pandemic.

## Results

### Overview

Our database search strategy yielded a total of 93 articles. A total of 80 articles were excluded for reasons of lack of fit with inclusion criteria, and two additional articles were identified from forward and backward reference searching. The result of the screening and supplementary reference search was a final sample of 15 included articles. Table 2 summarizes the included articles addressing virtual care and health equity during the COVID-19 pandemic, and Table 3 summarizes the findings from each included article according to the following three levels: (1) policy and government, (2) organization and health system, and (3) community and patient.

**Table 2:** Description of articles addressing virtual care and health equity during the COVID-19 pandemic.

Reference	Framing	Issues raised	Recommendations
Das and Gonzalez, 2020 [23]	Health care equity (equity in access to health services) is especially important to consider during COVID-19.	Access to technology (phones, phone lines, devices for virtual care) Digital literacy Cultural and linguistic issues Mistrust in health care systems	Select phone over video for certain populations Offer telemedicine outside of usual business hours Identify reimbursement models with insurers for underserved or marginalized patients Promote virtual widely to grow awareness among underserved or marginalized communities Partner with community organizations to provide peer-led technical support
Beaunoyer et al, 2020 [24]	Digital inequalities as a determinant of health. Suggest that digital inequalities enhance susceptibility to contracting COVID-19.	Outline 4 proximal influences on whether people can use technology: Technical means (the quality of the equipment that one can access, both in terms of hardware and software as well as the power and reliability of internet connection) Autonomy of use (the location where technology is accessed, and perceived freedom to use it as wanted) Social support networks (assistance from other experienced users) Experience (time dimension enabling people to be familiar enough with the technology to retain benefits from its use)	Increase access to connected devices Increase digital literacy (eg, educational programs) Increase access to relevant social support (eg, social support phone lines, user-friendly apps, etc) Increase diffusion of public health messages (eg, increase redundancy of important messaging) Increase control over quality of messaging Increase understandability of messaging Increase acceptability of messaging
Crawford and Serhal, 2020 [12]	Health equity; digital health innovation should not exacerbate existing health inequities during COVID-19.	Links between broader social determinants of health and the digital determinants of health Access to digital resources Use of digital resources for health seeking Digital health literacy Beliefs about potential for digital health to be helpful or harmful Values and cultural norms or preferences for digital resources Integration of digital resources into community and health infrastructure	Equal access to digital health leading to equal outcomes across identity groups Health providers trained to have competencies to provide equitable digital health care Measurement of equity-related outcomes Quality improvement focused on equity-related outcomes Involvement of people from marginalized groups in leadership, health professions, co-design, and data stewardship
Rodriguez et al, 2020 [15]	Digital divide should be considered in the implementation of recent policy (The 21st Century Cures Act)	Uptake of digital health tools is lower among marginalized populations Digital health tools have not been designed for marginalized populations	Promote access to broadband internet and digital devices Develop programs to promote digital health literacy Vendors should use inclusive design strategies Adopting organizations should embed equity in newly established digital services Offer digital services to all patients Government policy should clarify standards for design of digital health innovations
Eberly et al, 2020 [14]	Empirical evaluation of difference between those who completed scheduled telemedicine visits and those who did not. Vulnerable patients may have increased barriers to telemedicine care.	Findings highlight unique challenges faced by women, those who were non–English speaking, and poorer patients	Interpretation services Translation of instructions Improve distribution of video-enabling devices to those unable to afford them Payment parity between insurers for video and audio visits
Nouri et al, 2020 [13]	Health equity; relying on telemedicine risks further exacerbating inequities as certain patient groups may experience less access to care.	Reduced access to digital health among people in the following groups: Rural populations Older adults Racial/ethnic minority populations Low socioeconomic status Limited health literacy Limited English proficiency	Identify disparities in access Explore potential improvements related directly to existing disparities in access Mitigate digital literacy and resource barriers Remove health system barriers (offer video visits to every patient, ensure interpreter services, screen for patient barriers to video visits, offer telephone visits if video visits unavailable) Increase system leadership awareness of barriers to telemedicine Advocate changes to support equitable access (enable access to low-cost or free internet, pay parity for telephone and video visits from all payers)
Gray et al, 2020 [25]	Prevent exacerbation of health disparities	Adverse consequences of the digital divide most prominently affect low-income, rural, disabled, racial/ethnic minority, and older adult populations Sociocultural barriers to digital health: limited electronic skills, low health literacy, disability, low income, and limited English proficiency Structural barriers to digital health: geographic isolation, broadband capacity, and technical hardware Lack of touch also negatively affects communication with patients	Expand broadband access Accommodate language, literacy, and disability Provide telehealth literacy training Engage community health workers Promote digital empathy and webside manner
Ramsetty and Adams, 2020 [26]	Disparities in access to telemedicine care among vulnerable patients.	Lack of access to internet Cultural expectations of technology and its use in health care Mistrust of health care or of technology Literacy regarding digital technologies and digital health Lack of access to relevant digital devices Health care systems favoring newer, more expensive technologies	Combine technology and in-person visits, enabling care for people without access to technology (focused primarily on raising awareness about the digital divide during the pandemic)
Egan, 2020 [27]	An explicit focus on informal carers (known as unpaid caregivers in other contexts) and the challenges of engaging carers via digital health and virtual care.	A large proportion of carers have some form of disability A large proportion of carers use digital technologies Currently very few digital or virtual care initiatives are targeted toward caregivers in particular	Attention should be paid to providing virtual or digital resources specifically for caregivers
Friis-Healy et al, 2020 [28]	Increasing reliance on digital technologies risks exacerbating the digital divide, with adverse consequences on mental and behavioral health, especially of racialized populations.	Systemic racism and the pandemic are exacerbating mental health concerns for racialized communities, especially Black and Indigenous communities	Invest in building real-world evidence for digital mental health Educate providers and consumers about choice and safety of digital mental health Prioritize adaptive digital mental health content, allowing tailoring to particular communities Build digital mental health apps and services for diverse patient populations Build trust by evaluating and vetting in transparent ways
Jackson et al, 2020 [29]	The postpandemic future will see digital technologies dominating health spaces. Public health goal setting must attend to equity in digital health, particularly related to the vision of Health People 2030.	Persistent disparities exist in relation to internet access, using technology to manage health, online health information seeking, and health literacy	Ensure that health literacy and digital health objectives are a part of Healthy People 2030 Enhance data collection on digital health disparities Convene to critically discuss ideal objectives and strategies to achieve them
Kassamali et al, 2020 [30]	Policies enhancing access to telehealth services will expire at the end of the pandemic, but should persist for the sake of enhancing health equity.	Minoritized communities have had less access to health care during the pandemic Minoritized communities have been less able to shelter in place	Examine in detail how minoritized communities have adopted and engaged with telehealth services to inform equitable policy
Mike and Laroche, 2020 [31]	The pandemic has illustrated health inequities very clearly, and these extend to eye health as well. Short- and long-term actions are necessary.	Racism and structural inequalities are the causes of health inequities observed during the pandemic	More strongly incorporate telemedicine into eye care Advocate for policy changes that lead to insurance coverage for more people Take longitudinal action to address structural racism by encouraging cultural competence and holistic acceptance in medical education
Ortega et al, 2020 [32]	The pandemic has led to investments in telemedicine around the world. Specific policy considerations must be made to ensure telemedicine promotes health equity.	Inequitable access to telemedicine is driven by three main barriers: (1) disparities in access to broadband internet and related technology, (2) financial barriers to the reimbursement of telemedicine, and (3) lack of institutional commitment to equity in telemedicine	Policy must invest in expanding broadband internet access, enhance the availability of virtual care through reimbursement mechanisms, and clarify privacy and security requirements for commercially available platforms Hospitals should take on responsibility to enhance digital access and literacy
Wood et al, 2020 [33]	Many infectious diseases are disproportionately experienced by people from marginalized communities. The infectious disease community ought to invest in digital health equity.	Primary issues reducing access to virtual care are lack of technology, internet access, digital literacy, and private space in which to engage	Expanded reimbursement of telemedicine must continue after the pandemic Assess patient technical readiness. Provide just-in-time training to patients for access Provide instruction in preferred language Conduct a test to confirm capability Develop programs to offer digital devices to people who do not have access Offer language interpretation Design for various languages and cultural preferences Do not rely solely on electronic record–based portals for video visits Train clinical staff to consider equity when supporting patients virtually Track disparities in access and use disparities as a performance indicator

**Table 3:** Synthesis of recommendations from select literature on virtual care and health equity during the COVID-19 pandemic.

Level of initiative to enhance health equity in virtual care and general recommendations		Specific recommendations
**Policy and government**		
	Government policy	Government policy should clarify standards for inclusive design of digital health innovations Governments should increase access to relevant crisis and social services in support of marginalized communities (eg, social support phone lines) Governments should invest in maintaining expanded virtual care programs beyond the end of the pandemic
	Funder (reimbursement)	Identify reimbursement models with insurers for marginalized patients that can persist beyond the end of the pandemic Ensure payment parity between insurers for video and audio visits
	Access to devices and internet	Identify and document disparities in access to virtual care Promote access to broadband internet, especially among those who cannot afford it Promote access to digital devices among those cannot afford them (eg, through donations and lending programs at health care sites) Explore quality improvements related directly to existing disparities in access to digital devices
	Public health messaging	Increase emphasis on and diffusion of culturally relevant public health messages (eg, increase redundancy of important messaging) Increase control over quality, understandability, and acceptability of messaging about transmission, prevention, treatment, and consequences of COVID-19
**Organization and health system**		
	Organizational (health system or health care organization)	Measurement of equity-related outcomes such as number of visits using interpreter services Quality improvement focused on equity-related outcomes Train health providers to have competencies to provide equitable digital health care Increase virtual access and use of interpretation services for health care encounters Translate instructions for accessing virtual care Increase system leadership awareness of equity-related barriers to virtual care Offer telemedicine outside of usual business hours Promote virtual care widely to grow awareness among marginalized communities Adopting organizations should include equity considerations in newly established digital services Engage community health workers to provide technical support to patients with low digital literacy Provide interfaces in languages other than English Develop programs to lend digital devices to patients who do not have access to such devices during the course of care Provide access through a variety of programs, not solely through the electronic record system
	Clinical	Select phone over video for individuals who are not comfortable with video visits in the home environment Offer digital services to all patients Combine technology and in-person visits, enabling care for people without access to technology Advocate changes to support equitable access to virtual services at the local level Provide training and support to patients seeking to access care virtually Build processes for assessing patient readiness for virtual care Conduct test visits with patients for troubleshooting prior to scheduled virtual clinical visits
**Community and patient**		
	Community engagement in service planning and delivery	Partner with community organizations to provide peer-led educational support Involvement of people from marginalized groups in leadership, health professions, co-design, and data stewardship Vendors should use inclusive, user-centered design processes
	Enhance digital literacy	Develop programs to promote digital health literacy Mitigate digital literacy and resource barriers (eg, provide patient education to enhance digital literacy skills, inform patients about free or reduced-cost internet access locations)

### Recommended Strategies to Promote Health Equity

At the level of policy and government, recommendations have focused on strategies for health policy makers and health care funders to enable access both to the infrastructure required for patients to participate in virtual care (ie, inclusive design standards, broadband internet, and digital devices) and the availability of virtual care services to entire populations (eg, by appropriately reimbursing virtual care) [13,15,23,25,30-33]. In addition, policy-focused recommendations have emphasized the clarity of public health messaging about COVID-19 and related restrictions, and the role of digital technologies in enhancing the accuracy and reach of such messaging [24,29].

Recommendations at the level of health care organizations and health systems have been more varied. These have included encouragement to develop quality improvement activities focused on underserved or marginalized communities [12,18,27], educational initiatives for providers and leaders [12,23,29,33], and the collection of metrics that provide insight into equity-related outcomes [12]. Specific advice to clinicians has included strategies such as carefully planning a mix of in-person and virtual visits for clients especially at risk of poor health outcomes during the pandemic [25], and using telephone-based visits (over video visits) when a patient has access to a telephone but not a device that would enable a video visit [25,26,28,29].

At the level of communities and patients, recommendations focused on both the engagement of community members in service development and strategies to enhance digital literacy [13,25,26,29,30]. Specific approaches advocated include developing partnerships with community-based organizations and using inclusive design strategies that involve diverse users in the design of the technology [13,25]. Efforts to enhance digital literacy through particular educational programs during the pandemic were also common across the contributions we reviewed, including for example programs offered through local libraries [12,15].

Together, these recommendations provide a multilevel approach to ensuring that the widespread use of virtual care during the pandemic does not exacerbate disparities in access to care and health outcomes. In the next section of this paper, we outline three strategies that received relatively little attention in the reviewed literature We emphasize these three in particular because they are practically implementable by local health systems and have high potential for impact. Furthermore, these strategies are critical to the sustenance of equitable virtual care beyond the COVID-19 pandemic.

## Discussion

In this discussion section, we describe three specific strategies to promote health equity in the delivery of virtual care programs, and then outline three lines of action at the level of health system strategy to ensure these approaches are sustainable in the longer term.

### Simplify User Interfaces and Clinical Workflow

The first strategy that we highlight pertains to simplifying interfaces and workflows associated with accessing and using virtual care. Mounting evidence suggests that when innovations such as digital technologies increase the complexity of health care processes, they are more likely to widen existing health disparities [16]. This is because patients with less education, lower income, and a higher burden of negative social determinants of health (eg, food insecurity, precarious employment, etc) are less able to effectively integrate such innovations into their everyday lives or usual care [16]. This point is especially important given the ongoing financial and social challenges faced by marginalized communities during the COVID-19 crisis, which are likely to persist well beyond the end of the pandemic.

One reason for the elevated challenge of accessing and using complex virtual care technologies for marginalized patient groups relates to the technological infrastructure itself. For example, some technologies have high internet bandwidth requirements or are compatible with only a subset of expensive personal devices (eg, some video visit platforms do not run on Android devices). Virtual care strategies that work with simpler technology requirements are more likely to be accessible to people living with lower income, and are therefore more likely to be equity enhancing [16]. Such “upstream” strategies also have the potential to persist long term since they do not require agency on the part of providers or patients to maintain.

Additionally, significant digital literacy skills are necessary to benefit from virtual care technologies; marginalized groups with less technical experience, such as older adults and those of lower socioeconomic status, are less likely to have these skills [19,20]. Accordingly, virtual care platforms with usability challenges, high literacy demands, and complex workflows are more likely to benefit more advantaged users [16,20]. Thus, it is crucial that the design and delivery of technology-enabled services aim to minimize such barriers. This can be done by means such as using sequential rather than hierarchical navigation through the virtual care platform, an approach that reduces the cognitive burden associated with navigating through a computer interface by simplifying the information and number of choices presented to the user at any given time (ie, one choice at a time rather than a list of choices presented in a hierarchy). A second strategy is to reduce context switching, which occurs when the particular task in which the user is engaged (eg, booking a visit) is interrupted to perform a second task (eg, installing new software or opening a second program). More generally, following design guidelines for lower literacy populations (such as the guidelines produced by the United Nations Educational, Scientific and Cultural Organization [UNESCO] on designing inclusive digital solutions and developing digital skills [34]) will enhance the usability of any virtual care technology for all users. Where clinicians and organizations retain a degree of control over the content of virtual care technologies (more likely with larger health systems and organizations), these design-based solutions offer important strategies to promote health equity in the context of virtual care technologies.

### Use Supportive Intermediaries to Help Patients Engage With Virtual Care

In the context of the pandemic, many patients have been forced to engage with virtual care for the first time. In some sites, such as Federally Qualified Health Centers (FQHC) in the United States and other community-based services internationally, the infrastructure for virtual care might not have existed prior to COVID-19. This means that patients might not have had much opportunity to develop the skills required to engage with technology for their health care, especially among those facing other major financial- or health-related challenges. Moreover, first virtual care visits are not optimal environments for learning, fostering the patient-provider relationship, or managing chronic medical conditions as patients and providers are likely to have more urgent priorities related to addressing acute illnesses [35]. Additionally, reflective of the time pressure facing health care providers and staff, the last author’s (TV) experience with studying telehealth implementation in an FQHC shows that when challenges emerge during video visits, the immediate reaction is to switch to telephone calls. This results in far more telehealth visits being implemented via telephone than intended.

To address these challenges, we emphasize the strategy of integrating supportive intermediaries or liaisons within virtual care programs to assist new virtual care users in navigating visits. Human-computer interaction and sociology researchers have explored the use of intermediaries to bridge difficulties in access to and use of technologies among people with limited digital access and skills [36,37]. These intermediaries ideally serve as “warm experts,” people with relatively advanced knowledge of technologies who are made readily available to support peoples’ use of technology in their daily lives [38]. These supportive intermediaries might be identified in a number of ways. For larger organizations with staff that can be redeployed, team members can be trained in navigating a particular technology and provide intermediation to patients remotely. This model has been employed at the hospital (Women’s College Hospital) that is the primary affiliation of the first author (JS) during the COVID-19 pandemic, wherein research staff have been redeployed to support patients as they connect virtually with their health care providers. A second option arises from the suggestion of Gray et al (2020) [25] to enlist the support of community health workers where such workers are active in local communities. Community health workers can engage community members who are seeking out health care and provide direct support to patients as they engage with their care providers virtually. Additional strategies that can be pursued to identify and support intermediaries include hiring new staff to take on the intermediary role in permanent positions to assist beyond the pandemic for the long-term sustainability of health equity, building partnerships with community-based organizations, and collaborating with public libraries or patient advocacy groups to establish intermediary support programs. The creation of new staff roles and partnerships represents an infrastructure that has the potential to remain in place after the COVID-19 pandemic.

### Engage Members of Marginalized Communities in Planning and Evaluating Virtual Care

As with any health care innovation, virtual care programs risk overlooking the cultural, linguistic, and economic realities of marginalized communities [16,39]. In these cases, the virtual care program is far less likely to be taken up by marginalized patients, which also increases the risk of worsening health disparities. This point was addressed in many of the papers we reviewed, which advocated for the inclusion of marginalized communities in technology design, virtual care program design, training of health care providers, leadership of virtual care programs, and governance of data arising from such programs [12,15,23,25]. In the remainder of this section, we specify these recommendations by focusing on two practical strategies in particular: establishing a community advisory committee, and evaluating the service from the perspective of marginalized groups.

One practical strategy to engage members of marginalized communities in virtual care program delivery is to establish a community advisory committee that represents the views of marginalized patients. Collaborating with trusted partners who are prominent members of particular racial/ethnic or geographic communities could promote meaningful input into the development and improvement of the virtual care program [39]. Methods derived from community-based participatory research can help to ensure meaningful engagement of marginalized communities. Such engagement and input could have the additional benefit of providing a clearer understanding of the impacts of the social determinants of health during the pandemic, clarifying where interventions that look beyond the individual patient to the entire community are needed. Such input can thereby help health care providers and organizations to better understand the shifting needs of their patient populations and enhance their planning in both short-term and long-term time frames.

Evaluation that addresses the needs of underserved communities requires the identification of measures that matter most to members of these groups. This could also be accomplished through receipt of valuable input from key stakeholders, including patients or a community advisory board [12]. Measures should assess both objective features (such as dropped calls or total time spent interacting with patients) and subjective features of virtual care programs (such as satisfaction with and trust in health care providers). This information can be used to inform future quality improvements to the programs to better serve these diverse patient populations, especially in times of crisis when resources are scarce.

### Sustainability of Equitable Virtual Care at the Health System Level

Based on the insights generated through our review of literature, we propose three important lines of action to promote health equity in the delivery of virtual care on a large scale and in a sustainable way beyond the pandemic. First, governments and policy makers will need to navigate the demand for investments in infrastructure related to broadband internet access and the availability of digital devices for those who do not have access. The literature reviewed in our paper calls for programs that make devices available to patients who need them to actively engage with virtual care, which constitutes a unique expense for health systems with unique considerations for implementation. Balancing investments in internet access and the availability of digital devices with the clear need for additional investments in public health and the upstream determinants of health will be a central challenge for governments in the years ahead.

Second, health systems and stakeholder groups will need to specify clinical processes and develop effective training for the clinical skills that underpin the equitable delivery of virtual care. This will also require investment in educational programs and curriculum change to enable health care providers to employ virtual care in equitable ways.

Third and finally, health systems will need to invest in developing organizational capacity in health equity as a long-term priority. Where organizations do not already have knowledge of how to deliver equitable care in general, they cannot be expected to deliver virtual care in equitable ways. The development of educational programs such as those focused on anti-racism and anti-oppression initiatives will contribute to enhancing the health equity knowledge and capacity of health care organizations overall. Doing so will build an important foundation for incorporating a stronger focus on health equity into virtual care initiatives well into the future.

### Conclusion

The literature on strategies to promote health equity in virtual care programs in the context of COVID-19 presents a strong and comprehensive vision for the ways in which multiple stakeholders can work together to prevent worsening health disparities during the pandemic. However, to ensure that virtual care is employed in ways that are equity enhancing in a post–COVID-19 reality, further work is required. Health system leaders, clinicians, and the research community will need to more deeply engage with the literature summarized in this paper, and shift attention to the practical implementation of these strategies in the longer term.

## Abbreviations

FQHC: Federally Qualified Health Center
